# Regulation of the transcription factor NF-κB1 by microRNA-9 in human gastric adenocarcinoma

**DOI:** 10.1186/1476-4598-9-16

**Published:** 2010-01-26

**Authors:** Hai-Ying Wan, Li-Min Guo, Tao Liu, Min Liu, Xin Li, Hua Tang

**Affiliations:** 1 Tianjin Life Science Research Center and Basic Medical School, Tianjin Medical University, Tianjin 300070, PR China

## Abstract

**Background:**

MicroRNAs (miRNAs) are a new class of naturally occurring, small, non-coding RNAs that regulate protein-coding mRNAs by causing mRNA degradation or repressing translation. The roles of miRNAs in lineage determination and proliferation, as well as the localization of several miRNA genes at sites of translocation breakpoints or deletions, have led to speculation that miRNAs could be important factors in the development or maintenance of the neoplastic state.

**Results:**

We showed that miR-9 was downregulated in human gastric adenocarcinoma. Overexpression of miR-9 suppressed the growth of human gastric adenocarcinoma cell line MGC803 cell as well as xenograft tumors derived from them in SCID mice. Bioinformatics analysis indicated a putative miR-9 binding site in the 3'-untranslated region (3'UTR) of the tumor-related gene NF-κB1 mRNA. In an EGFP reporter system, overexpression of miR-9 downregulated EGFP intensity, and mutation of the miR-9 binding site abolished the effect of miR-9 on EGFP intensity. Furthermore, both the NF-κB1 mRNA and protein levels were affected by miR-9. Finally, knockdown of NF-κB1 inhibited MGC803 cell growth in a time-dependent manner, while ectopic expression of NF-κB1 could rescue MGC803 cell from growth inhibition caused by miR-9.

**Conclusion:**

These findings indicate that miR-9 targets NF-κB1 and regulates gastric cancer cell growth, suggesting that miR-9 shows tumor suppressive activity in human gastric cancer pathogenesis.

## Background

In recent years, a new class of naturally occurring, small, non-coding RNAs called microRNAs (miRNAs) have been discovered in plants and animals [1-3]. Unlike protein-coding mRNAs, miRNAs are first transcribed as long primary transcripts in the nucleus, and then cleaved to produce stem-loop-structured precursor molecules (pre-miRNAs) by Drosha [4]. Pre-miRNAs are then exported to the cytoplasm, where the RNase III enzyme Dicer further processes them into mature miRNAs (~22 nucleotides). Mature miRNAs negatively regulate gene expression at the post-transcriptional level. Through specific targeting of the 3'-untranslationed regions (3'UTR) of multicellular eukaryotic mRNAs, miRNAs downregulate gene expression by degrading target mRNAs or repressing their translation [5,6]. Since miRNAs bind the 3'UTR of target genes through partial sequence homology, miRNAs could control as much as 30% of all protein-coding genes [7].

An accumulating body of evidence has revealed critical functions for miRNAs in diverse biological processes, such as proliferation, apoptosis, and cell differentiation [8,9]. Thus, deregulation of miRNA expression may lead to a variety of disorders including many types of cancers [10]. Previous studies have identified cancer-specific miRNAs in many kinds of cancers, including ovarian cancer [11], lung cancer [12], breast cancer [13], hepatocellular carcinoma [14], and brain cancer [15,16]. In addition, miRNA genes are frequently located near genomic breakpoints [16] or are affected by copy number alterations [17]. Also, factors involved in miRNA biosynthesis may be dysregulated in human tumors [18,19]. Furthermore, a recent study described aberrant hypermethylation as a mechanism for miRNA genes including miR-9 inactivation and downexpression in human breast cancer [20]. Meanwhile, another study provided evidence that miR-9 acts as a tumor suppressor gene in recurrent ovarian cancer [21].

Gastric cancer is the second most common cause of cancer death in the world [22], and adenocarcinoma is the most common type of gastric cancer. In this study, we detected differential expression of miR-9 in human gastric adenocarcinoma and adjacent normal tissues through quantitative RT-PCR, and hypothesized miR-9 as a tumor suppressor. Consistent with this hypothesis, we observed that overexpression of miR-9 inhibited the growth of the gastric adenocarcinoma cell line MGC803 *in vitro *and *in vivo*. Subsequently, we predicted and confirmed that the tumor-related transcription factor NF-κB1 was a direct target of miR-9 and was negatively regulated by miR-9 at the post-transcriptional level. Finally, in MGC803 cells, suppression of NF-κB1 expression by specific small interfering RNA (siRNA) could also inhibit MGC803 cell growth, while ectopic expression of NF-κB1 could rescue MGC803 cell from growth inhibition caused by miR-9.

## Methods

### Clinical Specimen and RNA Isolation

Nine pairs of gastric samples, including nine human gastric adenocarcinoma tissue samples and nine matched normal gastric tissue samples, were obtained from the Tumor Bank Facility of Tianjin Medical University Cancer Institute and Hospital and the National Foundation of Cancer Research (TBF of TMUCIH & NFCR) with patients' informed consent. The category of gastric samples was confirmed by pathological analysis. The large RNA and small RNA of tissue samples were isolated using *mir*Vana™ miRNA Isolation Kit (Ambion) according to the manufacturer's instructions.

### Cell Culture and Transfection

The human gastric adenocarcinoma cell line MGC803 was grown in RPMI 1640 (GIBCO) supplemented with 10% fetal bovine serum, 100 IU/ml of penicillin and 100 μg/ml of streptomycin, incubated at 37°C in a humidified chamber supplemented with 5% CO_2_. Transfection was performed with Lipofectamine 2000 Reagent (Invitrogen) following the manufacturer's protocol. Spiked RFP-expressing vector was used to monitor transfection efficiency.

### Construction of Expression Vectors

To construct the pcDNA3/pri-miR-9 (pri-miR-9) expressing vector, we first amplified a 386-bp DNA fragment carrying pri-miR-9 from genomic DNA using the following PCR primers: miR-9-sense, 5'-CGGAGATCTTTTCTCTCTTCACCCTC-3', and miR-9-antisense, 5'-CAAGAATTCGCCCGAACCAGTGAG-3'. The amplified fragment was cloned into the pcDNA3.1 (+) at the BglII and EcoRI sites.

To construct the pSilencer/si-NF-κB1 (si-NF-κB1) vector, a 70-bp double-strand fragment was obtained via annealing reaction using two single-strands designed by Deqor (at http://cluster-1.mpi-cbg.de/Deqor/deqor.html): NF-κB1-Top, 5'-GATCCCGCCTGAACAAATGTTTCATTTGGTCAAGAGCCAAATGAAACATTTGTTCAGGCTTTTTTGGAAA -3'; and NF-κB1-Bot, 5'-AGCTTTTCCAAAAAAGCCTGAACAAATGTTTCATTTGGCTCTTGACCAAATGAAACATTTGTTCAGGCGG -3'. The fragment was then cloned into pSilencer 2.1 at the BamHI and Hind III sites.

To construct the NF-κB1 ectopic expression vector, the whole coding sequence of NF-κB1 without the 3'UTR was amplified by PCR from cDNA library of MGC803 cells. The PCR primers were as follows: NF-κB1-sense, 5'-CGGAATTCACCATGGCAGAAGATGATCC -3'; and NF-κB1-antisense, 5'-GTCAGCTCGAGAAATTTTGCCTTCTAGAGGTC -3'. The amplified fragment was cloned into the pcDNA3.1 (+) at the EcoRI and XhoI sites.

### Cell Proliferation Assay

Cells were seeded in 96-well plate at 4 000 cells per well the day before transfection. The cells were transfected with pri-miR-9 or control vector at a final concentration of 5 ng/μl as described above. To detect the dose-dependent effects, we gradually increased concentration of pri-miR-9 from 0 ng/μl to 15 ng/μl. To examine the time-dependent effects of si-NF-κB1 on MGC803 cells, MTT assay was used to measure the viable, proliferating cells at 24, 48, and 72 h after transfection. The absorbance at 570 nm was measured using a μQuant Universal Microplate Spectrophotometer (Bio-tek Instruments).

### Colony Formation Assay

After transfection, cells were counted and seeded in 12-well plates (in triplicate) at 100 cells per well. Fresh culture medium was replaced every 3 days. Colonies were counted only if they contained more than 50 cells, and the number of colonies was counted from the 6th day after seeding and then the cells were stained using crystal violet. The rate of colony formation was calculated with the equation: colony formation rate = (number of colonies/number of seeded cells) ×100%.

### *In vivo *Tumor Xenograft Studies

To establish the stable miR-9-overexpression cell line and the control cell line, MGC803 cells were transfected with pcDNA3/pri-miR-9 (pri-miR-9) or pcDNA3 (control), followed by selection for 20-30 days in complete medium supplemented with 800 μg/ml of G418 (Invitrogen). Single colonies were picked and amplified, and the expression level of miR-9 was detected by real-time RT-PCR. The stable miR-9-overexpression MGC803 cells or control cells were inoculated with 4 × 10^6 ^cells per site bilaterally on the axillary fossae of female athymic nude mice aged 6-8 weeks. Tumor size was monitored by measuring the length and width with calipers, and volumes were calculated with the formula: (L × W 2) × 0.5, where L is the length and W is the width of each tumor. The mice used in this experiment were maintained under specific pathogen-free conditions and handled in accordance with NIH Animal Care and Use Committee regulations.

### MiRNA Target Prediction

MiRNA predicted targets were predicted using the algorithms TargetScan, PicTar, and miRBase Targets. To identify the genes commonly predicted by the three different algorithms, results of predicted targets were intersected using MatchMiner.

### Fluorescent Reporter Assay

The EGFP expression vector pcDNA3/EGFP was constructed as previously described [23]. The 3'-untranslated region of NF-κB1 mRNA containing the miR-9 binding site was amplified by PCR using the following primers: NF-κB1 sense, 5'-CGCGGATCCTCAACAAAATGCCCCATG-3'; and NF-κB1 antisense, 5'-CGGAATTCAGTTAAATCGAGAATGATTCAGGCG-3'. The amplified fragment was cloned into pcDNA3/EGFP at BamHI and EcoRI sites at downstream of the EGFP coding region. Also, four nucleotides at the miR-9 seed sequence binding site of the NF-κB1 3'UTR were deleted using PCR side-directed mutagenesis assay. The two additional primers used in the mutation were as follows: NF-κB1 MS, 5'-CCACACCGTGTAACAACCCTAAAATTCCAC-3'; and NF-κB1 MA, 5'-GGAATTTTAGGGTTGTTACACGGTGTGG-3'. The fragment of NF-κB1 3'UTR mutant was similarly cloned into the pcDNA3/EGFP at the same sites.

MGC803 cells were transfected with pri-miR-9 or control vector pcDNA3 in 24-well plates, and then with the reporter vector pcDNA3/EGFP- NF-κB1 3'UTR or pcDNA3/EGFP- NF-κB1 3'UTRmut on the next day. The vector pDsRed2-N1 (Clontech) expressing RFP was spiked in and used for normalization. The intensities of EGFP and RFP fluorescence were detected with Fluorescence Spectrophotometer F-4500 (HITACHI).

### Quantitative RT-PCR

To detect the relative level of NF-κB1 transcript, real-time RT-PCR was performed. Briefly, a cDNA library was generated through reverse transcription using M-MLV reverse transcriptase (Promega) with 2 μg of the large RNA extracted from gastric tissue samples or MGC803 cells. The cDNA was used for the amplification of NF-κB1 gene and the β-actin gene was used as an endogenous control for the PCR reaction. PCR was performed under the following conditions: 94°C for 4 min followed by 40 cycles of 94°C for 1 min, 56°C for 1 min, 72°C for 1 min. PCR primers were: NF-κB1 sense and NF-κB1 antisense were as above; β-actin sense, 5'-CGTGACATTAAGGAGAAGCTG-3'; and β-actin antisense, 5'-CTAGAAGCATTTGCGGTGGAC-3'.

To detect the mature miRNA level, stem-loop RT-PCR assay was performed [24]. Briefly, 2 μg of small RNA was reverse transcribed to cDNA using M-MLV reverse transcriptase (Promega) with the following primers: miR-9-RT, 5'-GTCGTATCCAGTGCAGGGTCCGAGGTGCACTGGATACGACTCATACAG-3'; and U6-RT, 5'-GTCGTATCCAGTGCAGGGTCCGAGGTATTCGCACTGGATACGACAAAATATGGAAC-3', which can fold to a stem-loop structure. The cDNA was used for the amplification of mature miR-9 and an endogenous control U6 snRNA for all PCR reactions. PCR primers were: miR-9-Fwd, 5'-GCCCGCTCTTTGGTTATCTAG-3'; U6-Fwd, 5'-TGCGGGTGCTCGCTTCGGCAGC-3', and a universal downstream primer Reverse, 5'-CCAGTGCAGGGTCCGAGGT-3'. PCR cycles were as follows: 94°C for 4 min, followed by 40 cycles of 94°C for 30 s, 50°C for 30 s, 72°C for 40 s. SYBR Premix Ex Taq™ Kit (TaKaRa) was used following the manufacturer's instructions, and the real-time PCR was performed and analyzed by 7300 Real-Time PCR system (ABI). All primers were purchased from AuGCT Inc.

### Western Blot

MGC803 cells were transfected and lysed 48 h later with RIPA lysis buffer and proteins were harvested. All proteins were resolved on 10% SDS denatured polyacrylamide gel and then transferred onto a nitrocellulose membrane. Membranes were incubated with anti-NF-κB1 antibody or anti-GAPDH antibody with blotto overnight at 4°C. The membranes were washed and incubated with horseradish peroxidase (HRP) conjugated secondary antibody. Protein expression was assessed by enhanced chemiluminescence and exposure to chemiluminescent film. Lab Works™ Image Acquisition and Analysis Software (UVP) was used to quantify band intensities. Antibodies were purchased from Tianjin Saier Biotech and Sigma-Aldrich.

### Statistical Analysis

Data are expressed as means ± standard deviation (SD), and *P *≦ 0.05 is considered as statistically significant by Students-Newman-Keuls test.

## Results

### Quantitative Analysis of miR-9 Expression in Human Gastric Adenocarcinoma

**Figure 1:**
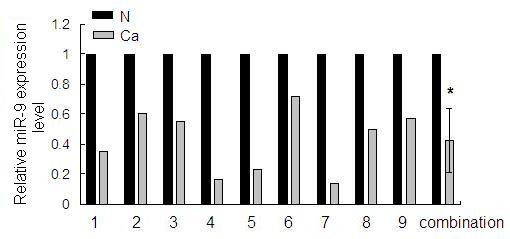
**Identification of differential expression of miR-9 in gastric tissues**. The expression level of miR-9 in the nine pairs of gastric adenocarcinoma tissues (Ca) and matched normal tissues (N) was detected by quantitative RT-PCR. U6 snRNA was regarded as an endogenous normalizer and the relative miR-9 expression level of the nine pairs of gastric tissues as well as the combined result (mean ± SD) is shown (*P < 0.05).

To test the expression of miR-9 in human gastric adenocarcinoma and adjacent normal tissues, real-time RT-PCR assay was performed in nine pairs of gastric tissue samples. It was shown that miR-9 expression level was generally and significantly lower in gastric adenocarcinoma tissues than in matched normal gastric tissues (figure 1).

### Suppression of Gastric Adenocarcinoma Cell Proliferation by Overexpression of miR-9 *in vitro*

**Figure 2:**
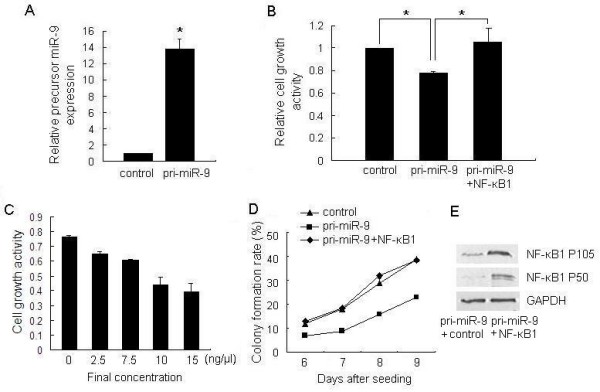
**Overexpression of miR-9 suppresses tumor cell growth *in vitro***. (A) In pcDNA3/pri-miR-9 transfected MGC803 cells, the mature miR-9 level was significantly increased. (B) MGC803 cells were transfected with pri-miR-9, control vector or pri-miR-9 with pcDNA3/NF-κB1, and cell growth activity was determined at 72 h post-transfection by MTT assay. Values are means ± SD of three duplications and the relative cell growth activity is shown (*P < 0.05). (C) MGC803 cells were transfected with gradually increasing concentrations of pri-miR-9 from 0 ng/μl to 15 ng/μl (final concentration) and the dose-dependent anti-proliferative effects were detected by MTT assay. (D) Cell independent growth activity was detected by colony formation assay. MGC803 cells were transfected with pri-miR-9, control vector or pri-miR-9 with pcDNA3/NF-κB1, and then seeded in 12-well plates. The number of colonies was counted from the 6th day after seeding. The colony formation rate was calculated and is shown. (E) The validity of NF-κB1 ectopic expression vector pcDNA3/NF-κB1 was confirmed by Western blot assay. MGC803 cells were transfected with pri-miR-9 as well as pcDNA3/NF-κB1 or control vector. The NF-κB1 protein level was detected by Western blot assay. GAPDH protein was regarded as endogenous normalizer and the relative NF-κB1 protein quantity is shown.

To determine the role of miR-9 in tumor cell proliferation, a miR-9 overexpression vector, pcDNA3/pri-miR-9 (pri-miR-9), was constructed. The validity of miR-9 ectopic expression was confirmed by quantitative RT-PCR, which revealed a 13-fold increase of miR-9 expression in pri-miR-9-transfected cells than in the control group (figure 2A). To test the effects of miR-9 overexpression on MGC803 cell proliferation, we investigated cell growth by MTT assay and found that pri-miR-9 could reduce MGC803 cell growth (figure 2B). Also, this effect showed a dose-dependent manner. At the concentration of 2.5 ng/μl and 15 ng/μl of pri-miR-9 plasmid, cell growth was inhibited by 15% and 45%, respectively (figure 2C). To further confirm the effect of miR-9 overexpression on the growth of gastric cancer cells, colony formation assay was performed. The colony formation rate of MGC803 cells transfected with pri-miR-9 was significantly lower than the control group (figure 2D). These results indicated that overexpression of miR-9 showed an anti-proliferative effect.

### Overexpression of miR-9 Inhibits Xenograft Tumor Growth *in vivo*

**Figure 3:**
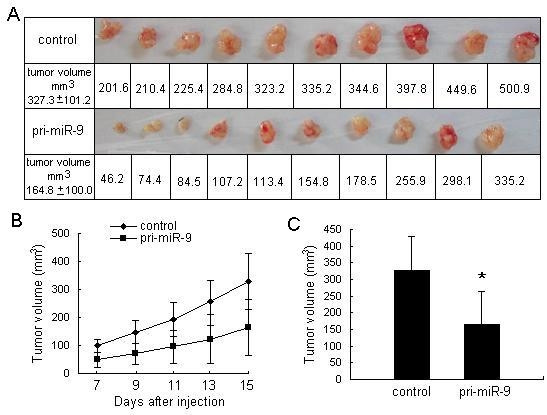
**Suppression of growth activity of xenograft gastric adenocarcinoma derived from MGC803 cells in SCID mice by overexpression of miR-9**. (A) The graphs show the differences between tumor volume in pri-miR-9 group and control group. (B) Tumor volume was measured after injection of MGC803 cells stably expressing pri-miR-9, and the tumor volume growth curve was drafted. (C) The difference of tumor volume between the pri-miR-9 group and control group was statistically significant (*P < 0.05).

To further determine whether miR-9 is involved in tumorigenesis, we established a stable miR-9-overexpression cell line as well as a control cell line. Cells of either line were injected into the axillary fossa of SCID mice, and the tumor growth activity was measured. Tumors derived from pri-miR-9-treated MGC803 cells grew substantially slower compared with the control group during the whole tumor growth period (figure 3B). When tumors were harvested, the average volume of tumors derived from the pri-miR-9 group was only half of that in the control group (figures 3A &3C). These results were consistent with the effects of miR-9 overexpression *in vitro *and strongly suggested that miR-9 could inhibit gastric cancer cell growth.

### NF-κB1 is a Candidate Target Gene of miR-9

MiRNAs can function as tumor suppressors or oncogenes, depending on whether they specifically target oncogenes or tumor suppressor genes [25-27]. Tumor suppressive miRNAs, such as miR-9, are usually underexpressed in tumors and may fail to control some of the oncogenic genes. Although underexpression of miR-9 in some types of tumors suggested its role in cancer development, the underlying mechanism is still unclear because little is known of miR-9 targets. Therefore, identification of miR-9-regulated targets is a necessary step to understand miR-9 functions. We used a three-step consequential approach to identify miR-9 target genes. First, target genes were predicted by bioinformatics analysis; second, target genes were validated by fluorescent reporter assay; and finally, the correlation of miRNA expression and target protein expression were detected to confirm the specific miRNA:mRNA interactions. The target genes of miR-9 were predicted using three programs, known as PicTar, TargetScan, and miRBase Targets. Of the predicted target genes, the oncogene NF-κB1, whose mRNA 3'UTR contained a putative binding site of miR-9, was identified. This analysis is consistent with the model in which tumor suppressor miRNAs inhibit tumor development by targeting and negatively regulating oncogenes.

### NF-κB1 Carries a Functional miR-9 Binding Site

**Figure 4:**
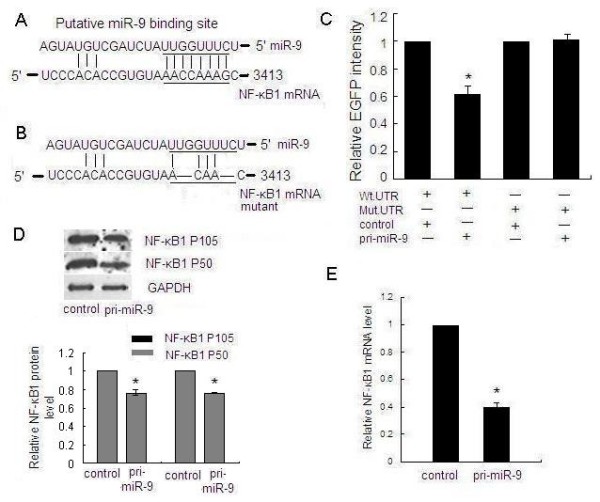
**NF-κB1 is a direct target of miR-9**. (A) The predicted miR-9 binding site on NF-κB1 mRNA 3'UTR is shown. (B) Deletion mutation at the miR-9 "seed region" binding site on the NF-κB1 mRNA 3'UTR is shown. Four nucleotides were deleted on the mutated 3'UTR. (C) MGC803 cells were transfected with the wild type of EGFP- NF-κB1 3'UTR (Wt. UTR) or mutated EGFP- NF-κB1 3'UTR (Mut. UTR) reporter vector as well as pri-miR-9 or control vector. Although pri-miR-9 suppressed the EGFP fluorescence intensity of EGFP- NF-κB1 3'UTR, mutation of the miR-9 binding site abolished the effect of miR-9 on the EGFP fluorescent intensity. (D) MGC803 cells were transfected with pri-miR-9 or control vector, and the NF-κB1 protein (P105 and P50) expression level was evaluated by Western blot. GAPDH protein was regarded as endogenous normalizer and the relative NF-κB1 protein quantity is shown. (E) MGC803 cells were transfected with pri-miR-9 or control vector, and expression of NF-κB1 mRNA was measured by quantitative RT-PCR. β-actin mRNA was regarded as an endogenous normalizer and the relative NF-κB1 mRNA expression level is shown. (*P < 0.05)

The mRNA 3'UTR of candidate miR-9 target gene NF-κB1 carries a putative miR-9 binding site (figure 4A). To confirm that this site was responsible for the negative regulation by miR-9, we cloned the putative 3'UTR binding site into the downstream of an enhanced green fluorescence protein (EGFP) reporter gene (EGFP-NF-κB1 3'UTR) and co-transfected this vector with pri-miR-9 or the control vector into MGC803 cells. The intensity of EGFP fluorescence of cells transfected with pri-miR-9 was decreased for about 1.7-fold, which was a statistically significant reduction compared with the control group (figure 4C). To further determine the function of the miR-9 binding site, we constructed another EGFP reporter vector containing the NF-κB1 3'UTR but with a mutated miR-9 binding site (figure 4B). As a result, pri-miR-9 had no effect on the intensity of EGFP fluorescence in this 3'UTR mutant vector (figure 4C), highlighting the importance of this miR-9 binding site. These results suggested that NF-κB1 is a direct target of miR-9.

### MiR-9 Negatively Regulates NF-κB1 at the Post-Transcriptional Level

**Figure 5:**
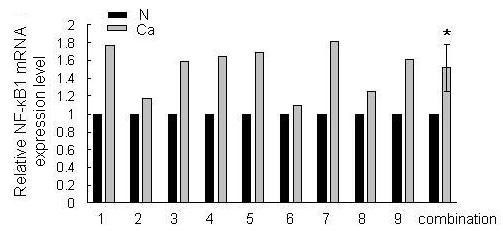
**Identification of differential expression of NF-κB1 in gastric tissues**. The expression level of NF-κB1 in the nine pairs of gastric adenocarcinoma tissues (Ca) and matched normal tissues (N) was detected by quantitative RT-PCR. β-actin mRNA was regarded as an endogenous normalizer and the relative NF-κB1 quantities of the nine pairs of gastric tissues as well as the combined result (mean ± SD) is shown (*P < 0.05).

Translational repression is regarded as a major mechanism for miRNA regulation of target gene expression [28]. To determine whether miR-9 suppresses endogenous NF-κB1 through translational repression, MGC803 cells were transfected with pri-miR-9 and the expression of NF-κB1 protein was examined by Western blot. It was shown that the amount of NF-κB1 protein was decreased after overexpression of miR-9 (figure 4D), suggesting that miR-9 negatively regulates endogenous NF-κB1 protein expression through translational repression mechanism. Furthermore, overexpression of miR-9 in MGC803 cells could also decrease the endogenous NF-κB1 mRNA level (figure 4E). In the nine pairs of gastric tissues, the NF-κB1 mRNA level showed a predominant up-regulation in gastric cancer tissues (figure 5). These data suggested that miR-9 negatively regulate the expression of NF-κB1 through mRNA cleavage mechanism.

### NF-κB1 Promotes Cell Proliferation and Independent Growth *in vitro*

**Figure 6:**
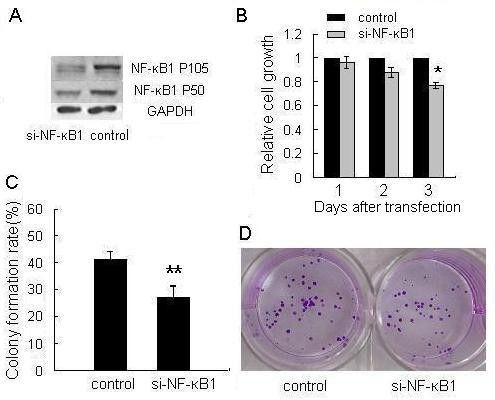
**Knockdown of NF-κB1 suppresses cell growth and colony formation**. (A) In pSilencer/si-NF-κB1 (si-NF-κB1) transfected MGC803 cells, the expression level of NF-κB1 protein was significantly decreased. (B) MGC803 cells were transfected with si-NF-κB1 or control vector. Relative cell growth activity was determined by MTT assay at the indicated times. Values are means ± SD of three duplications and the relative cell growth activity is shown (*P < 0.05). (C) Cell independent growth activity was detected by colony formation assay. MGC803 cells were transfected with si-NF-κB1 or control vector at a final concentration of 5 ng/μl (final concentration) and then seeded in 12-well plates. The number of colonies was counted on the 9th day after seeding. The colony formation rate was calculated and is shown (**P < 0.01). (D) The cells of colony formation assay were stained by crystal violet and the representative pictures of colonies are shown.

Previous studies have shown that the up-regulation of NF-κB1 is an important feature of many transformed cells, and that NF-κB1 functions as an oncogene. Accordingly, we detected whether NF-κB1 affects MGC803 cell growth. We constructed the pSilencer/si-NF-κB1 (si-NF-κB1) plasmid and confirmed that the expression of NF-κB1 protein was effectively suppressed by the siRNA expression vector (figures 6A). Then, the effect of NF-κB1 knockdown on cell growth was evaluated by MTT assay. As a result, suppression of NF-κB1 decreased cell growth in a time-dependent manner. At 72 h after transfection, we observed that cells transfected with the NF-κB1 knockdown vector grew more slowly than the control group (figure 6B). Also, the colony formation rate of the cells transfected with si-NF-κB1 vector was markedly lower than the control group (figures 6C &6D). Moreover, ectopic expression of NF-κB1 could rescue MGC803 cell from growth inhibition caused by miR-9, both in MTT assay (figure 2B) and colony formation assay (figure 2D). The validity of pcDNA3/NF-κB1 vector was confirmed by Western blot assay (figure 2E). These data, consistent with the previous findings [29], provided further evidence that NF-κB1 is an oncogene. The oncogenic role of NF-κB1 in gastric cancer may explain why overexpression of miR-9 can inhibit gastric cancer cell growth.

## Discussion

In the last few years, several studies have shown the dysregulation of miRNAs in various types of cancers [30-32]. Identification of cancer-specific miRNAs and their targets is critical for understanding their role in tumorigenesis and may be important for defining novel therapeutic targets [33-35]. Here, we focused on the role of miR-9 in the pathogenesis of human gastric adenocarcinoma. First, we examined miR-9 expression in gastric adenocarcinoma and matched normal gastric tissues by real-time RT-PCR assay as previously described [24]. This method uses a stem-loop reverse transcription primer and specially designed PCR primers to ensure the specificity of miRNA amplification. Meanwhile, U6 snRNA was also detected for normalization of expression in different samples. We discovered that from a total of nine pairs of matched advanced gastric adenocarcinoma tissue samples, the level of miR-9 was downregulated in tumor tissues compared to the matched normal tissues.

Several studies on the role of miR-9 deregulation in human oncogenesis have been reported. The aberrant hypermethylation study was done using the comprehensive methylation analysis approach, with breast cancer, normal breast tissues, and breast cancer cell lines as references. They found that the epigenetic inactivation of miR-9-1 in human breast cancer was due to aberrant hypermethylation, and found a strong correlation between hypermethylation of miR-9-1 and concomitant downregulation [20]. Interestingly, taking advantage of miRNA expression analysis and real-time TaqMan PCR, it was also found that miR-9 expression was decreased in recurrent ovarian cancer tissues compared to primary cancer tissues [21]. In addition, miR-9, miR-148a, and miR-34b/c were also underwent specific hypermethylation-associated silencing in cancer cells compared with normal tissues [36]. Hence, the high frequency of aberrant regulation of miR-9 in different types of cancer tissues and cells suggests that downregulation of miR-9 might play an important role in oncogenesis.

It has been presumed that miRNAs suppressed in cancers may normally function as tumor suppressor genes. Therefore, we hypothesized that miR-9 is a growth inhibition factor in human gastric adenocarcinoma. Since miR-9 expression is decreased in cancer tissues, we expected that overexpression of miR-9 would result in the arrest of cell growth. Using the MTT assay, we found that MGC803 cells transfected with the miR-9 overexpression vector (pri-miR-9) exhibited decreased growth compared to control cells. We also found that the anti-proliferative activity of pri-miR-9 transfection was dose-dependent. Cell independent growth activity, which is inconspicuous in normal cells, is a typical characteristic of malignant transformed cells. In colony formation assay, we observed that the colony formation activity of MGC803 cells transfected with pri-miR-9 was significantly inhibited. Furthermore, we found that the growth rate of tumors derived from MGC803 cells transfected with pri-miR-9 in SCID mice was lower than that of control tumors. Hence, these results indicate that gastric adenocarcinoma cells transfected with pri-miR-9 showed deletion of malignant phenotypes, suggesting a role for miR-9 in the growth suppression of cancer cells.

It was unclear as to how miR-9 affects cell growth and proliferation, because little is known about the physiologic targets of miR-9. Although bioinformatic tools may help to reveal putative mRNA targets of miRNAs, experimental procedures are required for their validation. Only a few studies have identified oncogenes whose level of expression is regulated by miRNAs. For instance, members of the let-7 miRNA family can negatively regulate all three members of the RAS oncogene family [27], and miR-15a/miR-16-1 can target and regulate BCL2 in B-cell CLL cells [25]. These findings support the idea that miRNA dysregulation may be involved in cancer pathogenesis. In this study, we show that miR-9 targets the NF-κB1 mRNA, thus revealing a potential mechanism associated with gastric tumorigenesis.

Experimental evidence indicated that NF-κB1 is a target of miR-9. First, the ability of miR-9 to regulate NF-κB1 expression is likely direct, because it binds to the 3'UTR of NF-κB1 mRNA with complementarity to the miR-9 seed region. Second, the EGFP fluorescence intensity of EGFP- NF-κB1-UTR was specifically responsive to miR-9 overexpression. Third, mutation of the miR-9 binding site abolished the effect of miR-9 on the regulation of EGFP fluorescence intensity. Fourth, we observed an inverse correlation between the expression of miR-9 and NF-κB1 in gastric adenocarcinoma tissues. Finally, endogenous NF-κB1 expression, both mRNA and protein, is decreased in pri-miR-9-treated MGC803 cells, suggesting that miR-9 may regulate NF-κB1 protein expression by inducing mRNA degradation and/or translational suppression.

The NF-κB1 gene encodes a 105 kD protein, which can undergo cotranslational processing by the 26S proteasome to produce the 50 kD protein. In fact, NF-κB1 is a member of the Rel/NF-κB transcription factor family, which plays important roles in the regulation of immune responses, embryo and cell lineage development, cell-cycle progression, inflammation, and oncogenesis [37-39]. Moreover, the positive rate of NF-κB1 in diffuse large B-cell lymphoma (DLBCL) was 63% (39/62), but none was expressed in reactive proliferation lymph node. However, NF-κB1 can positively regulate the expression of VEGF in DLBCL [40]. In addition, suppression of NF-κB expression can downregulate the expression of VEGF, thereby inhibiting the invasion and metastasis of tumors [41]. In this study, knockdown of NF-κB1 suppressed the growth of MGC803 cells, which was consistent with the results of miR-9 overexpression. Ectopic expression of NF-κB1 could also rescue MGC803 cells from growth inhibition caused by miR-9. However, the underlying mechanisms by which NF-κB1 affects gastric cancer cell growth remain to be established.

## Conclusions

In summary, we show that miR-9 expression is decreased in gastric adenocarcinoma tissues and that pri-miR-9 inhibits gastric cancer cell growth *in vitro *and *in vivo*. Furthermore, the expression of NF-κB1 can be negatively regulated by miR-9. This study extends our knowledge about the regulation of NF-κB1, a tumor-related protein. Thus, in addition to epigenetic regulation, NF-κB1 is also regulated at the post-transcriptional level by miRNA. It may suggest that miR-9 plays important roles in diverse biological processes by regulating NF-κB1.

## Competing interests

The authors declare that they have no competing interests.

## Authors' contributions

LMG and HYW performed the experimental work. ML and HT conceived of the study and participated in its design and coordination. XL analyzed and interpreted the data. TL helped to draft the manuscript. All authors read and approved the final manuscript.
